# Intervertebral disk width in dogs with and without clinical signs of disk associated cervical spondylomyelopathy

**DOI:** 10.1186/1746-6148-8-126

**Published:** 2012-07-28

**Authors:** Steven De Decker, Ingrid MVL Gielen, Luc Duchateau, Holger A Volk, Luc ML Van Ham

**Affiliations:** 1 Royal Veterinary College, University of London, Department of Veterinary Clinical Sciences, Hawkshead Lane, North Mymms, AL97TA, UK; 2 Ghent University, Faculty of Veterinary Medicine, Department of Medical Imaging of Domestic Animals and Orthopaedics of Small Animals, Salisburylaan 133, Merelbeke, 9820, Belgium; 3 Ghent University, Faculty of Veterinary Medicine, Department of Physiology and Biometrics, Salisburylaan 133, Merelbeke, 9820, Belgium; 4 Ghent University, Faculty of Veterinary Medicine, Department of Small Animal Medicine and Clinical Biology, Salisburylaan 133, Merelbeke, 9820, Belgium

**Keywords:** Cervical spondylomyelopathy, Wobbler syndrome, Magnetic resonance imaging, Morphometry, Intervertebral disk

## Abstract

**Background:**

Disk-associated cervical spondylomyelopathy (DA-CSM) is a multifactorial neurological disorder in which progressive caudal cervical spinal cord compression is mainly caused by one or more intervertebral disk protrusions. The Doberman pinscher breed seems predisposed for this condition. The underlying cause and pathophysiology of DA-CSM are currently unknown. Recently, wider intervertebral disks have been put forward as a risk factor for development of clinically relevant DA-CSM. However, little is known about other factors affecting intervertebral disk width. Therefore the aim of this study was to assess the association between intervertebral disk width, measured on magnetic resonance imaging (MRI), and clinical status, age, gender and intervertebral disk location in dogs with and without clinical signs of DA-CSM.

**Methods:**

Doberman pinschers with clinical signs of DA-CSM (N=17),clinically normal Doberman pinschers (N=20), and clinically normal English Foxhounds (N*=*17), underwent MRI of the cervical vertebral column. On sagittal T2-weighted images, intervertebral disk width was measured from C2-C3 to C6-C7. Intra –and interobserver agreement were assessed on a subset of 20 of the 54 imaging studies.

**Results:**

Intervertebral disk width was not significantly different between Doberman pinschers with clinical signs of DA-CSM, clinically normal Doberman pinschers or clinically normal English Foxhounds (p=0.43). Intervertebral disk width was positively associated with increasing age (p=0.029). Each monthly increase in age resulted in an increase of disk width by 0.0057mm. Intervertebral disk width was not significantly affected by gender (p=0.056), but was significantly influenced by intervertebral disk location (p <0.0001). The assessed measurements were associated with a good intra –and interobserver agreement.

**Conclusions:**

The present study does not provide evidence that wider intervertebral disks are associated with clinical status in dogs with and without DA-CSM. Instead, it seems that cervical intervertebral disk width in dogs is positively associated with increase in age.

## Background

Cervical spondylomyelopathy, also referred to as canine wobbler syndrome, is a multifactorial neurological syndrome that generally affects large and giant breed dogs [1]. Many lesions have been attributed to this syndrome and as a consequence, many synonyms can be found in the literature [2]. Over years, a few separate entities have been recognised based on clinical presentation and imaging findings [3,4]. Probably the most common of these entities is disk associated cervical spondylomyelopathy (DA-CSM) or disk associated wobbler syndrome [5]. In DA-CSM, caudal cervical spinal cord compression is mainly caused by protrusion of one or more intervertebral disks [5]. This disk associated spinal cord compression is sometimes seen in combination with dorsal compression resulting from hypertrophy of the ligamentum flavum and rather mild vertebral abnormalities such as flattening of the cranioventral aspect of the vertebral body and craniodorsal tilting of the vertebral body into the vertebral canal [5]. The intervertebral disk spaces between the sixth (C6) and seventh (C7) and/or the fifth (C5) and sixth cervical vertebrae are most commonly affected [6,7]. Disk associated cervical spondylomyelopathy occurs in several adult to older large breed dogs. The adult Doberman pinscher is overrepresented in several studies [6,8,9].

Although several factors have been proposed, the underlying pathogenesis of DA-CSM remains unknown [1]. Several morphometric studies have suggested pre-existing relative vertebral canal stenosis as a risk factor for development of DA-CSM [10-13]. Morphometric dimensions of the cervical vertebral column of Doberman pinschers with and without clinical signs of cervical spondylomyelopathy have been compared [11-14]. One of these studies demonstrated wider intervertebral disks and a narrower vertebral canal in clinically affected Doberman pinschers [11]. Therefore, it was suggested that the combination of a relatively stenotic vertebral canal with wide intervertebral disks could be a key feature in the pathogenesis of cervical spondylomyelopathy in Doberman pinschers [11].

However, other factors that could potentially influence intervertebral disk width such as age, gender, breed, and intervertebral disk location have not yet been investigated in dogs. Therefore, the aims of this study were to evaluate the influence of age, gender, intervertebral disk location, and clinical status on intervertebral disk width.

## Methods

### Animals

Fifty-four dogs were prospectively investigated. The experiment was conducted in accordance with the guidelines of the Animal care committee of the University of Ghent (Approval numbers 2007/075, 2008/060, and 2008/091). Written owner consent was obtained prior to study enrolment. Three groups of dogs were studied. The first group consisted of 17 client-owned Doberman pinschers with clinical signs of DA-CSM. This group consisted of 6 males and 11 females, between 4.4 and 10 years old (median, 7.0 years). Clinical signs varied from cervical hyperesthesia (N=3) to ambulatory paraparesis/ataxia (N=5), ambulatory tetraparesis/ataxia (N=7), and non-ambulatory tetraparesis (N=2). The second group consisted of 20 client-owned clinically normal Doberman pinschers. This group consisted of 11 males and 9 females, between 1.5 and 8 years old (median, 5.0  years). The third group consisted of 17 client (N=13) and laboratory-owned (N=4) English Foxhounds. This breed was included because of their comparable body conformation and body weight to Doberman pinschers and the fact that there is no known predisposition to DA-CSM in this breed. This group consisted of 9 males and 8 females, between 1.5 and 12 years old (median, 5.0 years).

In all dogs, physical and complete neurological examinations, complete blood cell counts, and serum biochemistry analyses were performed. All neurologic examinations were performed by the same person (SDD). All owners of the clinically normal dogs (N=37) were contacted at the end of the study period and encouraged to have another physical and neurologic examination performed on their dogs.

### Magnetic resonance imaging and measurements

**Figure 1:**
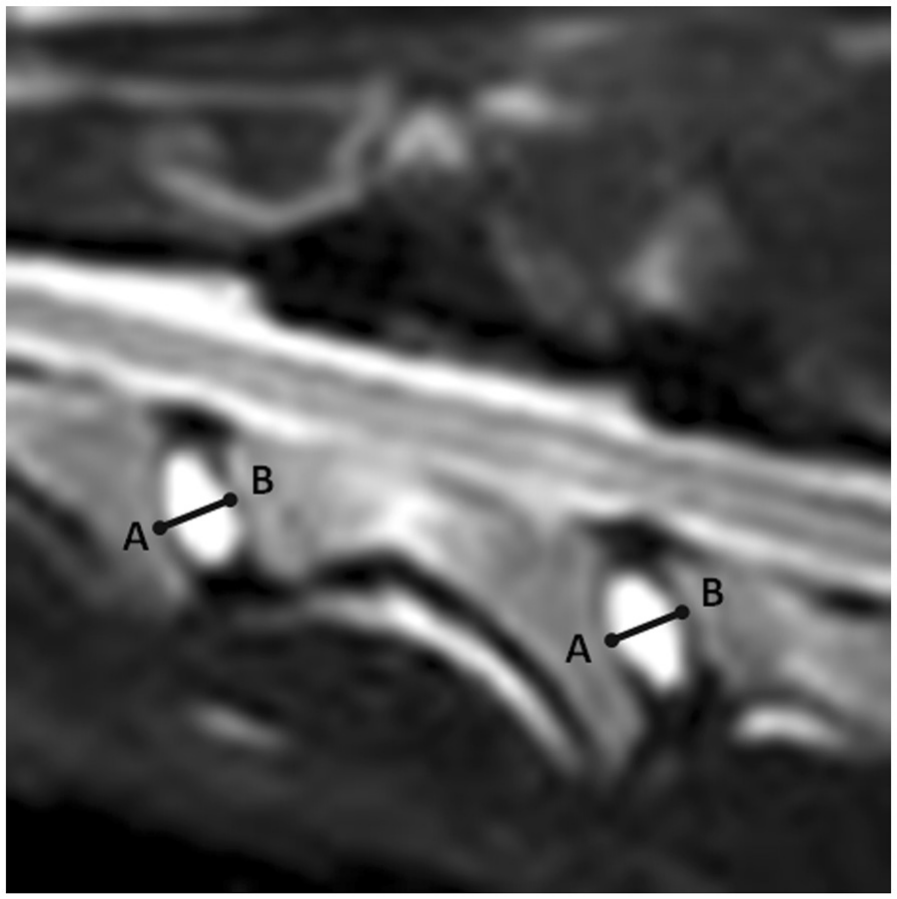
Intervertebral disk width (A-B) measured onmidsagittal T2-weigthed images

Cervical MRI under general anaesthesia was performed in all dogs. Dogs were anaesthetised with propofol and isoflurane in oxygen. A permanent, 0.2 Teslamagnet (Airismate, Hitachi) was used in all dogs. Dogs were positioned in dorsal recumbency with head and neck extended. The cervical vertebral column was positioned in a joint coil with an inner diameter of 19cm. For this study, only sagittal T2-weighted fast spin echo studies from C2 to C7 were assessed. The matrix size was 256 × 256 and the field of view was 29cm. Slice thickness was 4mm with no interslice gap. All MRI studies were evaluated by the first author (SDD). Measurements were made directly at the work station with Osirix image processing software. Measurements were recorded to 0.001cm. Intervertebral disk width was measured from C2-C3 to C6-C7 on the midsagittal T2-weighted images (Figure 1). Intervertebral disk width was measured at the widest point of the intervertebral disk as the distance of a line, originating at and perpendicular to, the cranial endplate of a vertebral body to the caudal endplate of the adjacent cranial vertebral body. To allow comparison with existing literature [11], intervertebral disks with imaging evidence of complete degeneration were excluded from the study. A completely degenerated disk was assumed to demonstrate a complete loss of hyperintense signal on midsagittal T2-weighted images.

Intraobserver agreement was tested by repeating the measurements on a subset of 20 out of 54 MRI studies by the first author (SDD). Interobserver agreement was tested by performing the same measurements on the same 20 different MRI studies by the second author (IG). These measurements were performed independently in a randomized sequence and both observers were blinded to signalment and clinical status of the subjects. Only the first measurements from the first author were further used to determine intervertebral disk width in the different groups of dogs.

### Statistical analysis

Intervertebral disk width was analysed by a mixed model with dog as random effect and clinical status, age, breed, gender, and intervertebral disk location as fixed effects. Global significance level was set at 5%; significance level for multiple comparisons was adjusted by Bonferroni’s method. Data were summarized by their means according to dog group. To evaluate intra and interobserver reliability, the mean difference, standard deviation (SD) of the difference and lower –and upper limits of agreement (LOA) were calculated.

## Results

A total of 270 intervertebral disks were assessed in 54 dogs. Thirty-seven (14%) of these 270 intervertebral disks were excluded from data analysis because of imaging findings suggestive of complete disk degeneration. Nineteen intervertebral disks were excluded in the group of clinically affected Doberman pinschers; thirteen of these disks were situated between C6-C7, 3 between C5-C6 and 1 each between C2-C3, C3-4, and C4-C5. Eight intervertebral disks were excluded in the group of clinically normal Doberman pinschers; six of these disks were situated between C6-C7 and 2 between C2-C3. Ten intervertebral disks were excluded in the group of clinically normal English Foxhounds; 8 of these were situated between C6-C7, and 1 each between C4-C5 and C5-C6. Significantly more intervertebral disks were excluded in the group of clinically affected dogs than in both groups of clinically normal dogs (p*=*0.0067). Twenty-seven of the 37 (73%) excluded intervertebral disks were situated between C6 and C7.

When looked at the average of all intervertebral disks, intervertebral disk width was not significantly different between Doberman pinschers with clinical signs of DA-CSM (mean: 5.58mm; ± SD 1.23mm), clinically normal Doberman pinschers (mean: 5.77mm; ± SD 1.21mm) or clinically normal English Foxhounds (mean: 5.54mm; ± SD 1.18mm) (p=0.42). Pair wise comparisons did not demonstrate any significant differences between clinically affected and clinically normal Doberman pinschers, between clinically affected Doberman pinschers and clinically normal English Foxhounds, or between clinically normal Doberman pinschers and English Foxhounds (Table 1). Even when compared for each intervertebral disk site separately (C2-C3 to C6-C7), no significant differences were present between the different groups of dogs (Table 1). Intervertebral disk width was positively associated with increasing age (p=0.029). Each monthly increase in age resulted in an increase of disk width by 0.0057mm. Intervertebral disk width was not significantly influenced by gender (p=0.0564). Intervertebral disk width was significantly influenced by the intervertebral disk location (p<0.0001). The overall intervertebral disk width between C2-C3 was significantly smaller compared to all other assessed intervertebral disks (Table 2). The overall intervertebral disk width between C3-C4 was significantly smaller than the overall intervertebral disk width between C4-C5 (Table 2).

**Table 1:** P-values of pairwise comparisons for measured intervertebral disk width between clinically affected Doberman pinschers, clinically normal Doberman pinschers, and clinically normal English Foxhounds for each assessed intervertebral disk space

**Group of dog**	**Overall**	**C2-C3**	**C3-C4**	**C4-C5**	**C5-C6**	**C6-C7**
Clinically affected vs.clinically normal DP	0.350	0.276	0.415	0.716	0.278	0.303
Clinically affected DP vs.clinically normal EFH	0.860	0.507	0.901	0.484	0.626	0.864
Clinically normal DP vs.clinically normal EFH	0.220	0.061	0.313	0.707	0.525	0.286

No significant differences were noted. Significance level for multiple comparisons is adjusted by Bonferroni’s method. DP=Doberman pinscher, EFH=English Foxhound.

**Table 2:** Influence of intervertebral disk location on measured intervertebral disk width

**Intervertebral disk space**	**Mean width (in mm)**	**p-value C2-C3**	**p-value C3-C4**	**p-value C4-C5**	**p-value C5-C6**	**p-value C6-C7**
C2-C3	5.36	NA	0.0045*	<.0001*	<.0001*	0.0008*
C3-C4	5.53	0.0045*	NA	0.0019*	0.0207	0.0561
C4-C5	5.79	<0.0001*	0.0019*	NA	0.438	0.728
C5-C6	5.72	<0.0001*	0.0207	0.438	NA	0.824
C6-C7	5.75	0.0008*	0.0561	0.728	0.824	NA

Mean overall intervertebral disk width and p-value for each assessed intervertebral disk space. Significant differences are indicated by (*). Significance level for multiple comparisons is adjusted by Bonferroni’s method.

Intraobserver agreement for the assessment of intervertebral disk width was considered acceptable. The mean intraobserver difference was – 0.00027mm (± SD 0.014mm) and was associated with a lower LOA of −0.27mm (5% of mean value) and an upper LOA of 0.24mm (4% of mean value). Interobserver agreement was also considered acceptable. The mean interobserver agreement was 0.0354mm (± SD 0.024mm) and was associated with a lower LOA of −0.44mm (8% of mean value) and an upper LOA of 0.51mm (10% of mean value).

Eighteen of 20 clinically normal Doberman pinschers and 9 of 17 English Foxhounds were available for physical and complete neurologic examinations between 16 and 18 months after the MRI examination of the study. These examinations revealed no abnormalities. The owner of four other English Foxhounds was available for a telephone interview 9 months after the MRI examination of the study. According to that owner, the dogs were clinically normal. The remaining 2 Doberman Pinschers and 4 Foxhounds died during the follow-up period for reasons unrelated to this study. According to the owners, these 6 dogs never had any clinical signs that were suggestive of a cervical myelopathy.

## Discussion

Although the authors of this study have already described the morphologic and morphometric cervical MRI findings of dogs with DA-CSM, clinically normal Doberman pinschers, and clinically normal English Foxhounds, no results have yet been reported on their intervertebral disk width [13-16]. These previous studies have demonstrated that the occurrence and degree of disk degeneration and protrusion could be considered part of normal age-related spinal degeneration [13,15]. Additionally, the present study suggests that cervical intervertebral disks in Doberman pinschers and English Foxhounds become wider with increasing age.

A recent study compared the morphometric features between 16 Doberman pinschers with clinical signs of cervical spondylomyelopathy and 16 clinically normal Doberman pinschers [11]. Significantly wider intervertebral disks in the group of clinically affected Doberman pinschers were demonstrated. It was suggested that the wider intervertebral disks of clinically affected Doberman pinschers could potentially be at higher risk of herniation and that the volume of disk protrusion into the vertebral canal would be higher than that of clinically normal dogs [11]. This finding, along with a relative vertebral canal stenosis, could potentially explain the development of clinical signs in Doberman pinschers with cervical spondylomyelopathy [11]. In contrast, the study reported here did not demonstrate that clinically affected Doberman pinschers have overall wider cervical intervertebral disks compared to clinically normal dogs. Even when looked at each intervertebral disk location separately, no significant differences were found between the different groups of dogs. Therefore, the results of this study do not provide evidence that wider intervertebral disks can be considered a potential risk factor for the development of clinical signs of DA-CSM. Although the exact reason is unknown, a potential explanation for the different results between both studies can be found in the age of included dogs. The mean age of the 16 clinically affected Doberman Pinschers in the study of da Costa et al. [11] was 6 years, while the mean age of the 16 clinically normal dogs was 4.3 years with only 3 dogs being older than 6 years of age. Since the results of the present study suggest that cervical intervertebral disks become wider with increasing age, it is possible that the difference found by da Costa et al. [11] could have been influenced, at least in part, by a difference in age between both groups of studied dogs.

The effects of advanced age on the imaging, morphometric, biomechanical and histopathological features of the intervertebral disk space have been investigated in human and laboratory-animal studies [17-23]. An altered fibrecohesivity [21], lamellae thickening, decrease in water and proteoglycan content with fragmentation and alterations in cross-linking of the collagen fibres in aged intervertebral disks has been demonstrated [19]. Such changes reduce the mechanical strength [19] and decrease the structural integrity of the aged disk [18]. These degenerative changes allow intervertebral disk deformation [21]. A study investigating age-related changes in the spine of laboratory-Beagles [17] demonstrated a decreased elastic modulus of the aged intervertebral disk along all levels of the vertebral column. The intervertebral disk spaces became less strong and less stiff with increasing age [17]. Although not significantly different, a recent study demonstrated higher values for cervical intervertebral disk thickness in 6 geriatric compared to 4 aged rats [23]. Unfortunately, this study was limited by a rather small population size and the morphometric variable “intervertebral disk thickness” was associated with a low statistical power. In contrast to our findings, increased age in people usually results in an age related narrowing of the intervertebral disk space [20]. This discrepancy is potentially related to different biomechanical properties of the bipedal human compared to the quadrupedal canine vertebral column. In rats, it has been demonstrated that bipedism and upright posture can change vertebral canal dimensions in the lumbar vertebral column [24].

No significant difference in intervertebral disk width was demonstrated between clinically normal Doberman pinschers and clinically normal English Foxhounds. This suggests that intervertebral disk width should not be considered a strictly breed specific morphometric feature. However, it should be emphasized that only two large-dog breeds with a comparable body conformation were included in this study. This is highlighted by the results of a recent study, demonstrating different micromorphometric variables between chondrodystrophic and non-chondrodystrophic dogs [25]. Therefore, further studies are indicated to assess intervertebral disk width in different dog breeds with a variable body conformation. Until such studies have been performed, we do not recommend extrapolating our results of intervertebral disk width to other dog breeds. Although not statistically different in our study population, there was a trend for male dogs to have wider intervertebral disks compared to female dogs. Larger body dimensions of male dogs compared to female dogs can probably explain this finding.

In this study, intervertebral disks with imaging findings suggestive for complete intervertebral disk degeneration were excluded from further analysis. This was done for several reasons. First, this was considered necessary to allow comparison of our results with the results of da Costa et al. [11], which currently represents the only similar study in veterinary literature. Although not strictly defined an exclusion criterion in that study,completely degenerated disks were not included for further analysis, as they could not be measured accurately [11]. Second, it has been suggested that intervertebral disk width cannot be reliably assessed on low-field MRI in case of complete intervertebral disk degeneration [9]. More specifically, it seems very difficult to discriminate the hypointense signals from the completely degenerated disk and the adjacent vertebral endplates [9]. Third, it cannot be excluded that complete intervertebral disk degeneration in itself would influence intervertebral disk width and act by this way as a confounding variable when comparing different groups of dogs. Not surprisingly, the majority of excluded intervertebral disks were situated between C6-C7. We cannot exclude that this could have influenced the results of our study at this specific anatomic level. The rather high proportion of excluded disks between C6-C7 can be explained by the fact that this site is most commonly affected in both naturally occurring DA-CSM [6,7] and age related disk degeneration in clinically normal large-breed dogs [11,15]. The predilection of the more caudally located cervical intervertebral disk spaces to demonstrate disk degeneration in large breed dogs is most likely related to the increased amount of axial rotation possible in the caudal cervical vertebral column compared to the cranial cervical vertebral column [26]. Axial rotation is facilitated by concave shaped articular facets and it has been demonstrated that the cervical vertebrae of large breed dogs have more pronounced concave shaped articular facets than small breed dogs [27]. Axial rotation is considered the main force responsible for disk degeneration,more so than flexion, extension, and lateral bending [28].

## Conclusions

In summary, the present study does not provide evidence that overall wider intervertebral disks are associated with clinical status in dogs with and without DA-CSM. Instead, cervical disk width is positively associated with increase in age. Age related changes should be considered when comparing morphometric features between different groups of dogs.

## Abbreviations

DA-CSM, Disk-associated cervical spondylomyelopathy; MRI, Magnetic resonance imaging.

## Competing interests

None of the authors of this paper has a financial or personal relationship with other people or organisations that could inappropriately influence or bias the content of the paper.

## Authors’ contributions

SDD drafted the manuscript, carried out the clinical assessments, and performed the measurements. IG performed the measurements for interobserver agreement and was responsible for performing the imaging studies. LD performed the statistical analysis and discussed the experimental design. HV helped to draft the manuscript. LVH helped to draft the manuscript and was the supervisor of this research project. All authors read and approved the final manuscript.
